# Global Hip Offset is an Important Factor in the Success of Abductor Mechanism Insufficiency Repair After Total Hip Arthroplasty: A Case Series

**DOI:** 10.1016/j.artd.2025.101861

**Published:** 2025-10-10

**Authors:** Samo Roškar, Neža Trebše, René Mihalič, Nejc Kurinčič, Mateja Blas, Rihard Trebše

**Affiliations:** a Valdoltra Orthopaedic Hospital, Ankaran, Slovenia; b Faculty of Medicine, University of Ljubljana, Ljubljana, Slovenia; c Arthroplasty Registry of Slovenia, Ankaran, Slovenia

**Keywords:** Abductor reconstruction, Abductor repair, Gluteus transfer, Whiteside procedure, Offset

## Abstract

**Background:**

Hip abductor mechanism deficiency due to abductor tendon degeneration, tear, or intraoperative damage during total hip arthroplasty (THA) may cause severe walking disability. For severe abductor weakness in native hip joints, Whiteside muscle transfer is a good solution. However, the literature on the results of abductor mechanism reconstruction (AMR) after THA remains limited. Our study aimed to assess the outcome of AMR in patients with THA suffering from Milwaukee III and IV hip abductor deficiency.

**Methods:**

We conducted a single-center retrospective cohort study of THA with hip abductor mechanism deficiency treated surgically with AMR. Data were collected between January 2011 and December 2019 and included the following parameters: patient’s data, subjective level of pain, Harris Hip Score (HHS), gait pattern, extent of hip abductor tear, and offset measurements.

**Results:**

The cohort included 16 THAs with AMR in 16 patients. The whole group median HHS improved from 37.1 interquartile range (IQR) (31.0-38.7) to 73.9 IQR (63.5-83.7) (*P* < .001). In a subgroup of 9 hips, the global offset was preserved after THA while it was reduced in remaining 7 hips. All patients with preserved global offset had significantly better clinical outcome compared to the group with reduced global offset (median HHS improvement was 48 IQR [46-53] compared to 22 IQR [18-25], *P* = .001).

**Conclusions:**

Our study showed favorable outcome of the AMR for chronic, Milwaukee III and IV hip abductor deficiency after THA. It is the first study to show that restoration of global offset after THA is of utmost importance to avoid chronic abductor mechanism deficiency.

## Introduction

The gluteus medius and minimus muscle-tendon complex (abductor mechanism) is crucial for normal gait and stability [1]. The damage of the abductor mechanism may result in major functional deterioration. Depending on the extent of abductor damage, patients suffer from various degrees of trochanteric pain, hip weakness, limp, positive Trendelenburg sign, and early tiring [2,3]. Severely affected patients require walking support [2,4,5]. Abduction weakness (AW) may present as a complication (muscle tear or abductor denervation) after total hip arthroplasty (THA), but it may also occur spontaneously due to abductor tears (ATs), spinal nerve damage, or different neurologic diseases. ATs are associated with tendon degeneration, overuse, or acute trauma [2,4,6]. Regarding the etiology, AW after THA may be iatrogenic due to intraoperative muscle and/or tendon damage, superior gluteal nerve damage, altered biomechanics, or a combination of lesions. It can also result from failed healing after surgical procedure. Poor implant selection and/or inferior THA biomechanics may further aggravate AW [[7], [8], [9]]. Degenerative tears could be associated as well to unfavorable hip-knee biomechanics [[10], [11], [12]]. ATs are present in 20% of patients undergoing THA concomitantly with hip joint degeneration [13]. AW after THA is one of the serious and difficult-to-treat complications resulting in late dislocations and to an unknown extent probably also in implant failure and spinal degeneration [12,14].

The first local muscle transfer using gluteus maximus was described by Bentzon in 1930 who presented the technique on two patients with poliomyelitis-related hip abductors deficiency [15]. The technique was revived by Whiteside in 2011 and modified in 2013 by adding the tensor fascia latae muscle flap to the transfer complex which resulted in significant reduction in limping and decreasing the proportion of postoperative Trendelenburg sign [16,17]. The reason for that could be that transferred muscle fibers align in the optimal direction for substitution of the deficient abductor muscles [18]. The transferred muscles provide static stability and allow for active hip abduction. They could hypertrophy and gain strength with time. Cortical control of the transferred muscle function and proprioception probably improves with time as well. Therefore, a long rehabilitation period is expected and proper physiotherapy seems important with final treatment results achieved only after one to 2 years of follow-up [[16], [17], [18]]. In addition to Whiteside abductor mechanism reconstruction (AMR) procedure, there are also other AMR techniques with good results. These include endoscopic direct repair [[19], [20], [21], [22]], direct transosseous reconstructions with sutures [23,24,25,26], and/or augmented grafts [3,23,24]. The comparison with open technique is difficult since the characteristics of the tears differ significantly.

The review of the literature shows a disproportionally small number of papers dealing with relatively frequent and debilitating abductor mechanism failure after THA [2,8,14,25,26]. However, there is no consensus on the procedure of choice for abductor mechanism insufficiency, and an optimal technique for surgical repair has not been defined yet. The initial conservative treatment involves activity modification, specific physical therapy, and analgesia [27,28]. For characterization of the extent of abductor damage, Milwaukee classification was introduced with four groups, where Milwaukee I correspond to 25%, Milwaukee II to 50%, Milwaukee III to 75%, and Milwaukee IV to 100% damage of abductors [29]. Among surgical options of AMR reconstruction, there are open and endoscopic AMR repairs, arthroscopic procedure seems to be more appropriate choice for smaller lesions (Milwaukee I and II), while the open procedure is more suitable for bigger lesions (Milwaukee III and IV) [3,14,[20], [21], [22],^25^,^26^,[30], [31], [32]].

The aim of this study was to assess the clinical results of hip AMR for severe Milwaukee III and IV hip abductor ruptures in THA using the modified Whiteside technique [17] performed through the open direct lateral approach. Second aim was to assess the impact of global offset change after THA on clinical outcome of AMR.

## Material and methods

We conducted a single-center, single-surgeon retrospective cohort analysis of a consecutive series of patients undergoing revision THA between January 2011 and December 2019. Only patients with chronic and severe hip abductor mechanism deficiency were included (Milwaukee III and IV). In total, 69% of patients had primary arthroplasty outside our institution. The prospectively collected patient comprised: age at the time of surgery, sex, body mass index (BMI), pain level on visual analog scale (VAS), Harris Hip Score (HHS), Trendelenburg sign, presence of limping, and global hip offset before and after primary THA.

The limping was classified in three groups based on its severity, the Trendelenburg sign test was performed in one-legged stance. A positive sign was recorded if the opposite hemipelvis sagged more than 2 cm.

The indications for surgery included one or a combination of the following signs and symptoms: persisting abductor-related disability despite conservative management, intolerable pain in the hip trochanteric region, limping, a positive Trendelenburg sign, and weakness of the resisted abduction of the hip. The neurological status of gluteus maximus was confirmed clinically by a palpable contraction and with an electromyography when needed. The abductor mechanism deficiency was additionally confirmed with ultrasonography and/or computed tomography or magnetic resonance imaging. ATs were categorized according to the Milwaukee classification into four groups (I = 25%, II = 50%, III = 75%, IV = 100%) [29].

**Figure 1:**
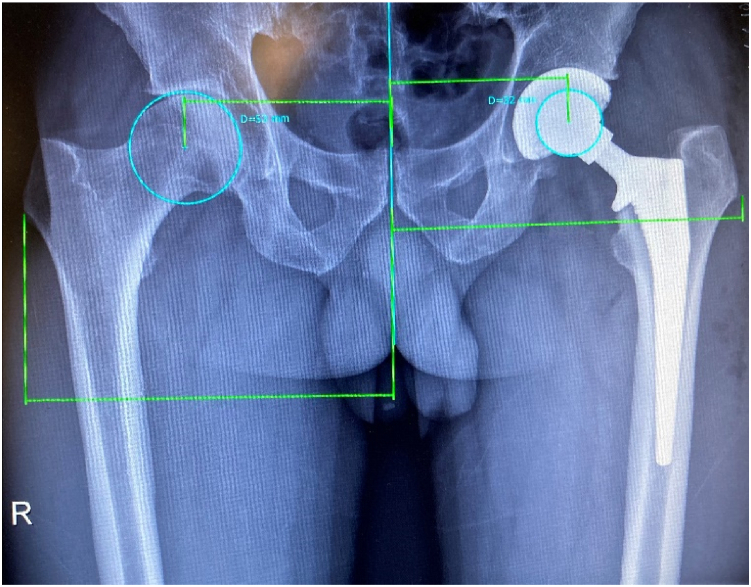
Offset measurement as described by Bjarnason and Reikeras.

Offset measurements were performed on standing anteroposterior plain hip radiographs using a special software (mediCAD Hectec GmbH, Altdorf, Germany). The hip global offsets were measured as described by Bjarnason and Reikeras (Fig. 1) [33].

### Surgical technique and postoperative rehabilitation

The surgery was performed with the patient in the lateral decubitus position with the ipsilateral leg in 30° of abduction and 30° of flexion in a leg rest. The trochanteric region was reached through a posteriorly extended lateral skin incision including preexistent scars when present. Proximal part of the incision was extended posteriorly for adequate gluteus maximus muscle exposure. Local gluteus maximus (and tensor fasciae latae occasionally) muscular flaps were then transferred on the greater trochanter in line with the technique published by Whiteside [34]. A detailed description of the entire surgical procedure is included in the Appendix.

The postoperative rehabilitation protocol included early partial weight bearing (up to 15kg) with two-handed support and avoidance of active or passive adduction and active abduction exercises for 6 postoperative weeks. Plaster cast with 20° of flexion and 20° of abduction was applied for 4-6 weeks in patients not able to comply with the suggested precautions. After this period, weight-bearing increases for 10 kg per week with concomitant abduction exercises in a standing position and abduction exercises against gravity were encouraged.

### Statistical analysis

Data were presented with absolute values and percentages or medians with interquartile range (IQR). Wilcoxon signed-rank test was used to test the differences before and after surgery and Mann-Whitney test for the between-group comparisons. The statistical analysis was performed using IBM SPSS Statistics version 29.0 for Windows (IBM Corp. Armonk, NY, USA). *P* < .05 was considered statistically significant.

## Results

Our study included 16 hips from 16 patients (9 females). The median age at the time of surgery was 67.8 IQR (65.0-75.0) years. The median time of follow-up was 26.8 IQR (17.5-30.7) months. The median whole group BMI of the patients was 28.4 IQR (26.7-31.7) kg/m2; among them 7 (44%) were obese (BMI >29.9 kg/m2). No patient was lost to follow-up and all were available for a clinical and radiological review. Complete, full-thickness tear (Milwaukee IV) was noted in 13 (81%) hips and more than 75% thickness tear (Milwaukee III) in 3 (19%) hips, no Milwaukee I and II tear were included. In all index surgeries, the lateral approach was used. In total, 69% of patients had primary arthroplasty (index surgery) outside our institution. The median time from the index surgery to AMR was 1.93 IQR (1.92-5.00) years. General characteristics of the cohort are summarized in Table 1.

**Table 1:** Main results of the analyzed cohort.

Whole cohort	
Rupture thickness (n [%])	
Full	13 (81%)
>75%	3 (19%)
Harris Hip Score (mean [IQR])	
Preoperative	37.1 (31.0-38.7)
Postoperative	73.9 (63.5-83.7)
VAS (mean [IQR])	
Preoperative	5.4 (4.0-7.0)
Postoperative	2.7 (1.7-4.0)
Positive Trendelenbourg sign (n [%])	
Preoperative	16 (100%)
Postoperative	7 (44%)
Subgroup with preserved offset	
Global offset (mean [mm])	
Preoperative	149
Postoperative	147
Mean difference Harris Hip Score (IQR)	48 (46-53)
Subgroup with altered offset	
Global offset (mean [mm])	
Preoperative	161
Postoperative	151
Mean difference Harris Hip Score (IQR)	22 (18-25)

**Figure 2:**
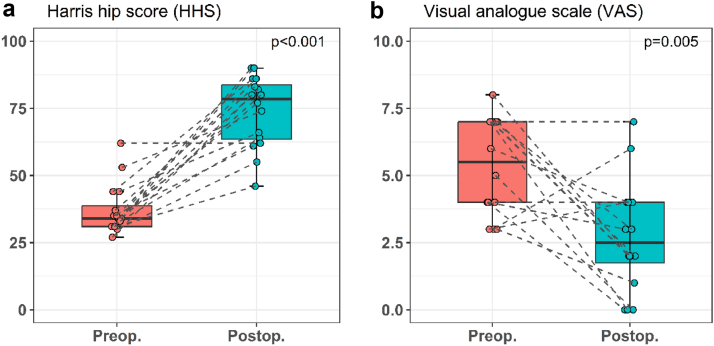
Change in Harris Hip Score (HHS) (a) and pain on visual analog scale (VAS) (b) for the whole cohort before and after Whiteside procedure.

The median whole group HHS improvement was significant, increasing from 37.1 IQR (31.0-38.7) preoperatively to 73.9 IQR (63.5-83.7) (Wilcoxon signed-rank test, *P* < .001) (Fig. 2a). The median whole group VAS score decreased from 5.4 IQR (4.0-7.0) to 2.7 IQR (1.7-4.0) (Wilcoxon singed-rank test, *P* < .001) (Fig. 2b). The number of hips with positive Trendelenburg sign decreased from 16 (100%) preoperatively to 7 (44%) postoperatively. Preoperatively, all patients were limping, 14 (87%) severely and 2 (13%) moderately. After surgery, 5 (31%) patients were limping severely, 7 (44%) patients moderately, and 4 (25%) patients had no limp.

**Figure 3:**
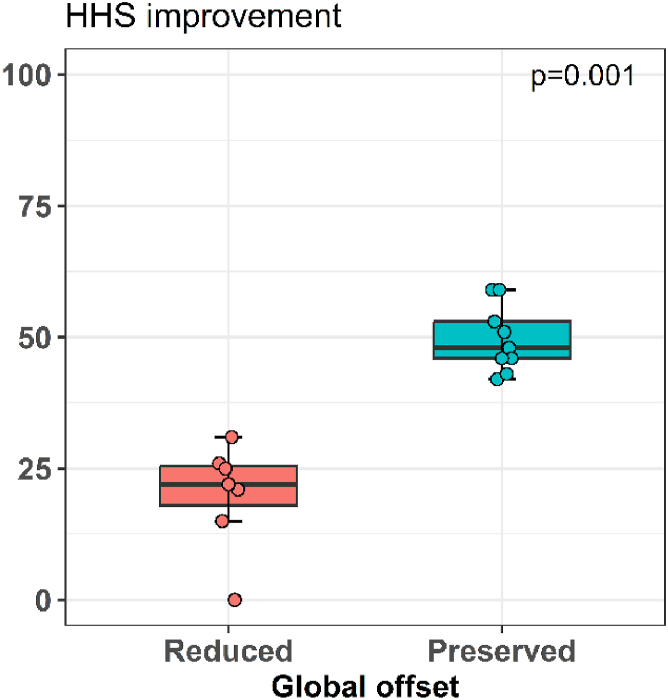
The change in Harris Hip Score (HHS) related to the offset of the hip joint.

**Figure 4:**
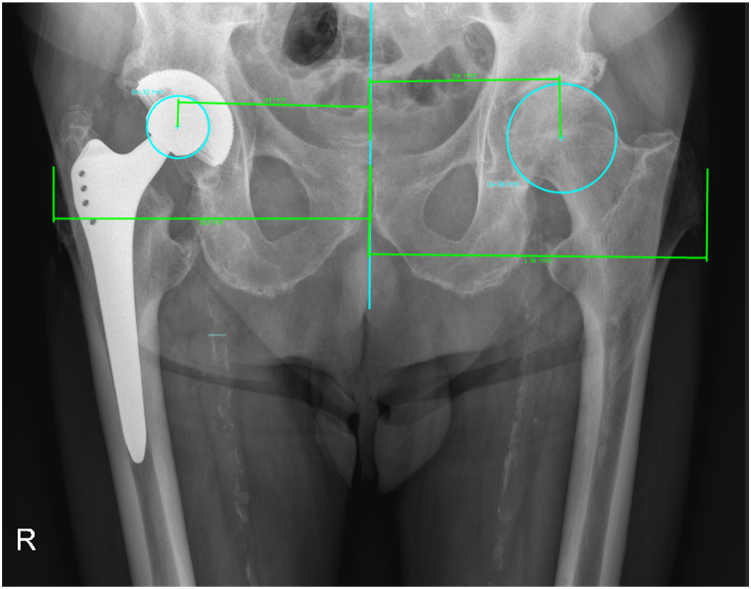
Decreased global offset situation after total hip arthroplasty on the right side.

During global offset measurement and result analysis after AMR, we identified a subgroup with excellent post-AMR result where the global offset was preserved with primary THA. This subgroup included 9 hips (7 females, median age of 68.8 years, median BMI 29.4 kg/m2) where the mean global offset before and after primary THA were 149 mm and 147 mm, respectively. The median increase in HHS was 48 IQR (46-53). The median VAS score decreased for 2 IQR (1-4). In the subgroup of 7 hips (7 patients, 4 females, median age of 66.6 years, median BMI 26.8 kg/m2) with less successful Whiteside procedure, the global offset was considerably decreased in all patients. We registered no patient with increased global offset. Among patients with less successful Whiteside procedures, the median global offset before THA was 161 mm and it decreased to 151 mm after primary THA. The median postoperative HHS increase was 22 IQR (18-25). The median VAS score decreased for 5 IQR (0-5). The difference in HHS increase in the preserved global offset group was significantly better compared to the altered offset group (Mann-Whitney U test; *P* = .001) (Fig. 3 and Fig. 4).

One AMR in the altered offset group failed due to trauma after the initial surgery and was successfully revised. One patient suffered from pulmonary embolism without long-term consequences. There were no other medical or surgical complications according to the standardized list of surgical complications developed by the Hip Society [35].

## Discussion

Our study shows a favorable outcome of the AMR with modified Whiteside procedure [17] in patients with THA suffering from chronic, Milwaukee III and IV hip abductor mechanism deficiency. In our case series of 16 AMR in THA patients, significant improvement of function and pain was observed in all cases at a median follow-up of 26.8 months (HHS improvement 36.8; VAS decrease for 2.7, 56% without Trendelenburg sign). The whole group results are in line with other publications reporting the results of gluteus maximus muscle transfer [16,17,[36], [37], [38], [39]].

In accordance with our findings, Christofilopoulos et al., using the Geneva technique, also reported on comparable results with significant reduction of limping, and of the Trendelenburg sign postoperatively [36]. Quisquater et al. observed absence of Trendelenburg sign in less than 50% of patients, but all had a significant improvement in pain score [37]. In our study, we found that 56% of patients after AMR procedure have no more positive Trendelenburg sign. Maldonado et al. reported that abductor strength improved in only 41% of patients, but on the other hand, there was a significant postoperative improvement in HHS and VAS [38]. In our study, we found significant improvement of HHS, 36.8 IQR (32.5-45.0) and VAS decrease, 2.7 IQR (2.3-3.0). Ruckenstuhl et al. reported on similar results to our study, they also observed improvement in Trendelenburg sign in less than 50% of patients, but their cohort showed no significant improvement in HHS and abductor strength. On the other hand, 72% of patients would undergo the same treatment again [39]. In contrast to these findings, we observed only one case of the need for the revision AMR procedure and it was due to trauma after the initial surgery.

Considering that our cohort includes only carefully selected patients with Milwaukee III-IV abductor rupture, it seems that chronic abductor muscle degeneration is the major impediment to the success of endoscopic or direct repairs [30]. This is in line with Maslaris et al., who found no differences in clinical outcomes between open and endoscopic approaches when patients were carefully selected for one or another procedure. Importantly, they treated more complex tears with an open approach, thereby introducing a significant bias. Nevertheless, they found that abductor degeneration adversely impacted outcomes [40].

Among different surgical techniques available for the AMR, the endoscopic procedures (direct repair, direct transosseous reconstructions with sutures and/or augmented grafts) are considerably less invasive methods of AMR treatment, with the major issue of considerably lower suture strength of the created construct compared to open techniques [[19], [20], [21]]. According to the literature available, endoscopic techniques are suitable for Milwaukee I and II ruptures [[19], [20], [21]]. Direct comparison of different endoscopic techniques with the open technique is thus difficult since the degree of the tears differs significantly. In general, open procedures are reported to be more appropriate for degenerative mostly full thickness tears, Milwaukee III and IV [16,17,23,24,26,27]. The direct transosseous abductor repair using nonabsorbable sutures to reattach the abductor mechanism to the greater trochanter is unpredictable and is usually not effective enough in restoring abductor function, or even detrimental [22,[30], [31], [32]]. The open transosseous repair of larger abductor tendon defects augmented by allografts also produce good results, but intact abductor muscle mass is needed [3,16,17,24]. Among different surgical techniques reported in the literature, the Whiteside procedure is associated with the lowest influence on the donor side of the flap compared to the proportion of Trendelenburg sign improvement [16,17,23,24,26,27].

Whiteside measured the offsets in his cohort of abductor repairs, but did not include any results based on this nor comments regarding the offset influence on the results [41]. In our study in the subgroup of 9 hips, where the global hip offset was preserved after primary THA, we observed significantly better results than in the subgroup of patients with an altered-decreased global offset (HHS improvement was 48 IQR [46-53] vs 22 IQR [18-25]; *P* = .001). A possible explanation for this phenomenon is the presence of unfavorable biomechanics after THA, particularly the decreased global hip offset. The situation influences hip biomechanics by reducing lever arm of abductor muscles producing less torque resulting in relative abductor muscle insufficiency with positive Trendelenburg sign and limping. As more abductor force is required for the same functional effect, increased muscle fatigue and degeneration develops.

The presented study has several limitations. It is a case series report and further studies on bigger cohorts are required. All surgeries were performed by single, high-volume surgeon using the same approach, which may affect the generalizability. The mean follow-up was 26.8 months, so we could only speculate on long-term results. Despite the weaknesses, according to our knowledge, the presented study is the first that indicates the importance of restoration of the global offset while performing THA in order to achieve a well-functioning abductor mechanism. The study also raises a concern if it is at all indicated to perform abductor repair without component revision in severe offset derangement.

## Conclusions

In summary, our study shows a favorable functional outcome of the AMR for chronic, Milwaukee III and IV hip abductor mechanism deficiency after THA. Local muscle transfer as described by Whiteside is a functional solution for hip AMR in Milwaukee III and IV grade tears with no late dislocations, implant failures, or any other major complications related to the surgical procedure. Moreover, our study is the first case series to show that restoration of the hip global offset after THA is of outmost importance for functional hip abductor mechanism. In patients with abductor mechanism insufficiency and altered global offset after THA concomitant restoration of the offset and the hip abductor mechanism repair should be considered.

## Conflicts of interest

R. Mihalič is an EFORT Board Member; R. Trebše serves on the Biomerieux Speakers bureau, is a Zimmer Biomet consultant, is an unpaid Ceramtech consultant, receives research support from Zimmer Biomet, receives other financial or material support from DePuy Sinthes, receives royalties from Springer Verlag, is an EBJIS Past President; all other authors declare no potential conflicts of interest.

For full disclosure statements refer to https://doi.org/10.1016/j.artd.2025.101861.

## Ethics approval

The study was approved by the Institutional Ethics Committee of the Valdoltra Orthopaedic Hospital (No. 15/2020). The study was performed in accordance with the ethical standards of the institutional research committee and with the 1964 Helsinki Declaration and its later amendments or comparable ethical standards.

## Informed patient consent

The author(s) confirm that written informed consent has been obtained from the involved patient(s) or if appropriate from the parent, guardian, power of attorney of the involved patient(s); and, they have given approval for this information to be published in this case report (series).

## CRediT authorship contribution statement

**Samo Roškar:** Writing – review & editing, Writing – original draft, Visualization, Software, Methodology, Investigation, Formal analysis, Data curation, Conceptualization. **Neža Trebše:** Writing – review & editing, Writing – original draft, Visualization, Methodology, Investigation, Formal analysis, Conceptualization. **René Mihalič:** Writing – review & editing, Writing – original draft, Visualization, Validation, Supervision, Software, Methodology, Investigation, Formal analysis, Data curation, Conceptualization. **Nejc Kurinčič:** Writing – original draft, Investigation, Formal analysis, Conceptualization. **Mateja Blas:** Writing – review & editing, Writing – original draft, Visualization, Validation, Software, Methodology, Formal analysis, Data curation, Conceptualization. **Rihard Trebše:** Writing – review & editing, Writing – original draft, Visualization, Validation, Supervision, Software, Resources, Project administration, Methodology, Investigation, Funding acquisition, Data curation, Conceptualization.

## Figures and Tables

**Figure S1:**
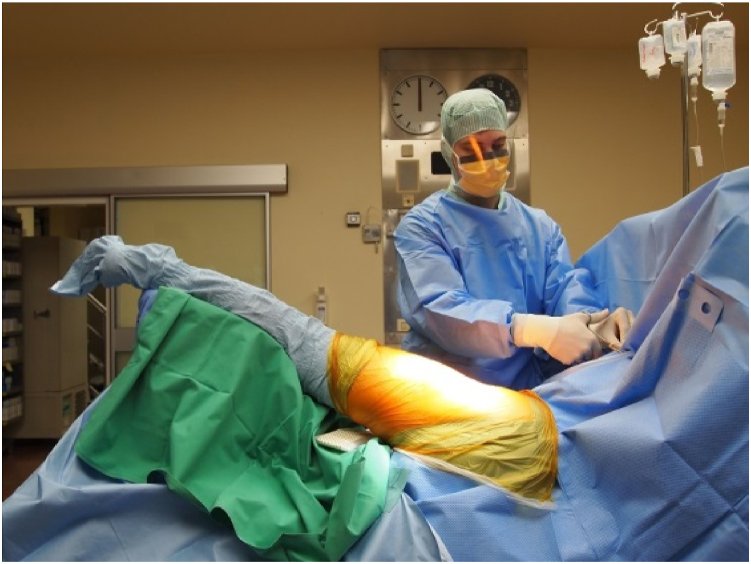
Patient positioning in the operating theater.

**Figure S2:**
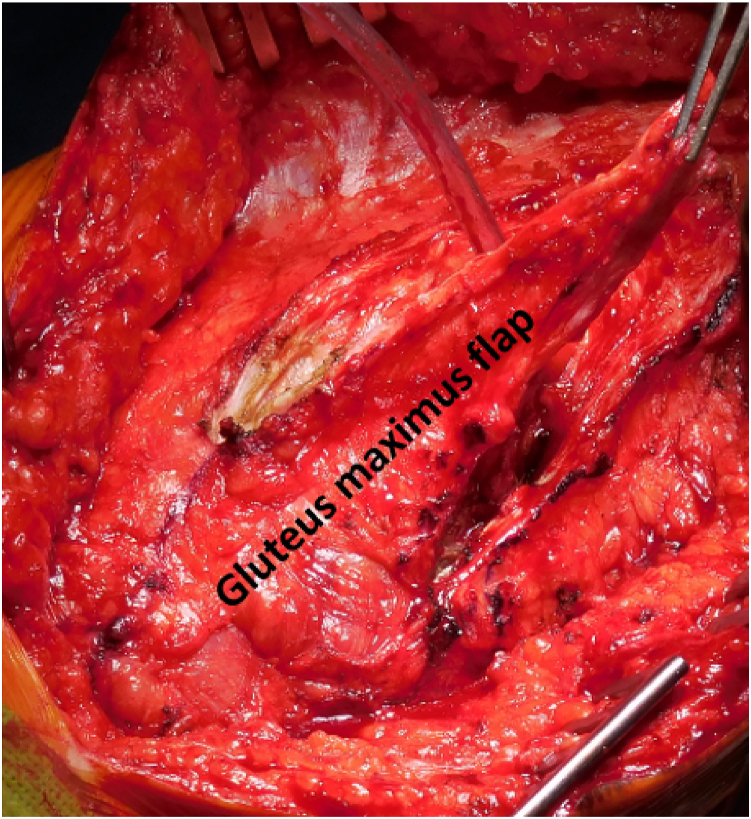
Preparation of the gluteus maximus muscle flap.

**Figure S3:**
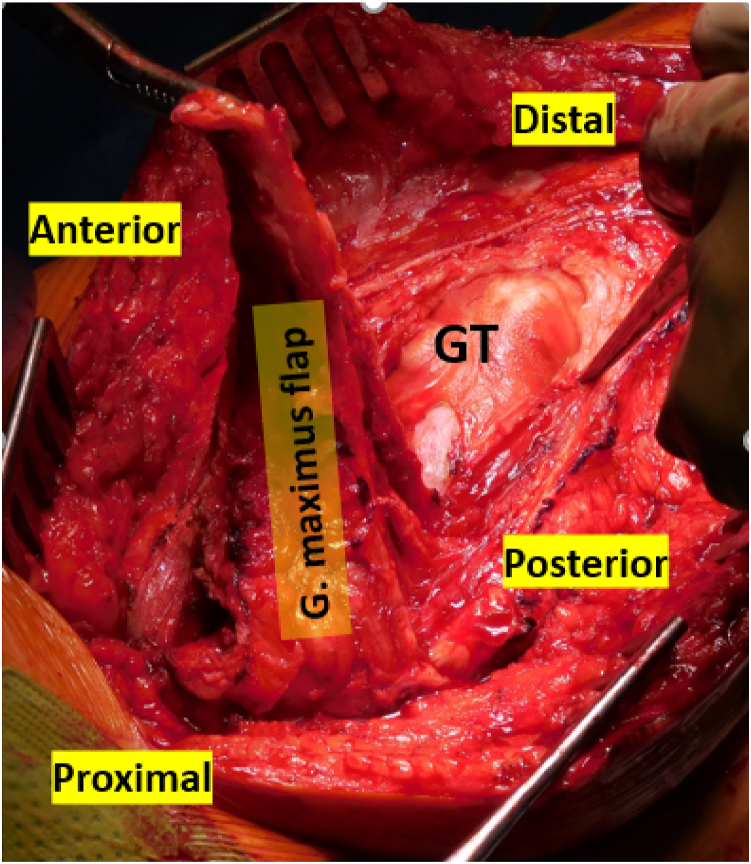
Decortication of insertion site on the greater trochanter.

**Figure S4:**
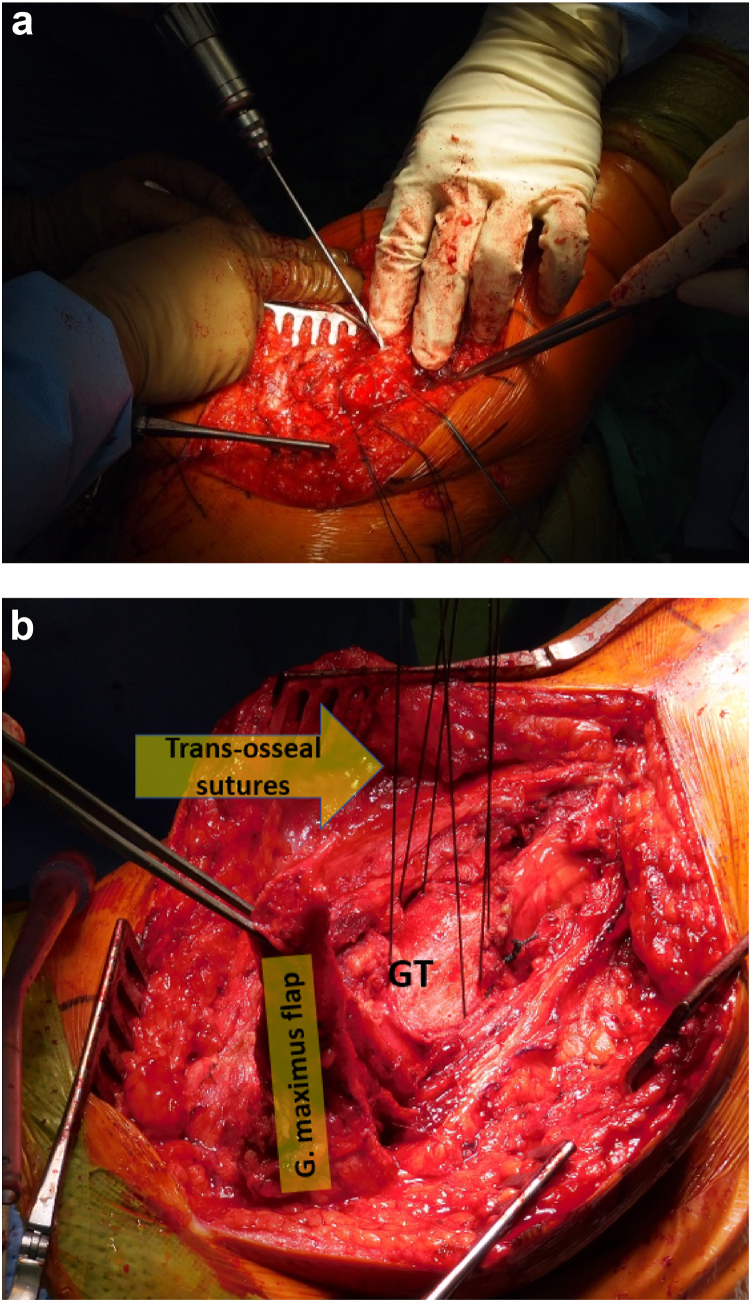
Drilling the holes to decorticated grater trochanter (a) Nonabsorbable sutures passed through predrilled holes for flap attachment (b).

**Figure S5:**
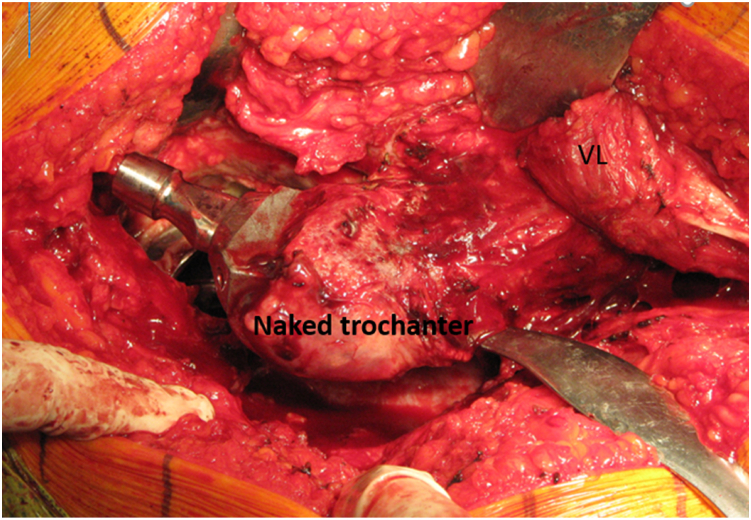
The site of the muscle flap transfer, completely naked greater trochanter.

**Figure S6:**
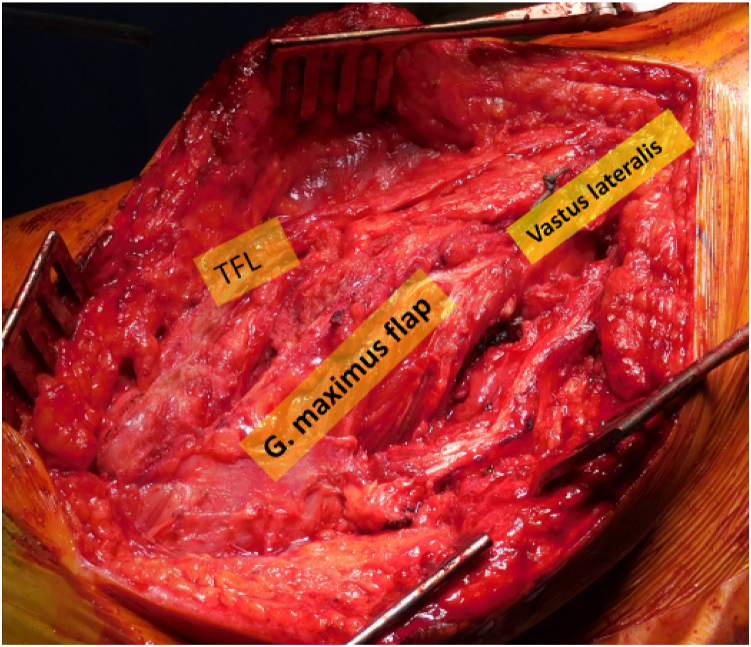
The final situation with the gluteus maximus fixed on the trochanter in line with vastus lateralis greater trochanter. TFL, tensor fascia latae.

## Appendix

### Detailed description of the surgical procedure

The surgery was performed with the patient on the lateral decubitus position with the ipsilateral leg in 30° of abduction and 30° of flexion in a leg rest (Fig. S1). The procedure starts by exposing the fascia lata followed by a straight incision along the planned anterior line of the gluteus maximus flap followed by bursectomy. The anterior third of the gluteus maximus is dissected with a 5 × 2 cm long extension of fascia latae (Fig. S2). The remnants of the abductors (to some extent present in most cases) are identified, released from adhesions, secured on a nonresorbable suture, and passed through a predrilled tunnel of the grater trochanter from anterosuperior to posteroinferior direction and securely fixed into the tunnel with transosseous sutures and into the external rotator tendons. A proximal (cca 5cm) dissection of the vastus lateralis from the bone is preformed followed by exposure of the bleeding bone on lateral side of the trochanter for flap attachment (Fig. S3). Four–6 holes are drilled trough the lateral trochanter, and the muscle flap is sutured to the trochanter under moderate tension (Fig. S4) in 30° abduction with strong nonabsorbable sutures (Fig. S4). Heavy, nonabsorbable sutures (№ 5 Ethibond, Ethicon Inc, Raritan, New Jersey, USA) are utilized for the flap attachment. The proximal part of vastus lateralis is advanced proximally and sutured over the gluteus maximus reinsertion on the trochanter to give fresh blood supply to the healing reconstruction. In cases with absolutely no tendon fibers attached to the greater trochanter down to below the level of the tuber innominatum (Fig. S5), a tensor fasciae latae flap is cross sutured over the gluteus maximus transfer on the greater trochanter. The distal fascia lata part of the flap is sutured trough the vastus lateralis (Fig. S6). The muscle transfer is covered by closing the fascia lata as proximal as possible with absorbable sutures (№ 3 Vicryl, Ethicon Inc, Raritan, New Jersey, USA) (Fig. S6) and the standard closure of the wound is performed.

## Appendix A Supplementary data

Conflict of Interest Statement for Trebše

Conflict of Interest Statement for Roškar

Conflict of Interest Statement for Mihalič

Conflict of Interest Statement for Blas

Conflict of Interest Statement for Kurinčič

Conflict of Interest Statement for Trebše
