# Supplementary material for: Global Hip Offset is an Important Factor in the Success of Abductor Mechanism Insufficiency Repair After Total Hip Arthroplasty: A Case Series
Source: Arthroplast Today. 2025 Oct 10;36:101861. doi: 10.1016/j.artd.2025.101861 (PMC12549797; doi:10.1016/j.artd.2025.101861)
Supplement: Conflict of Interest Statement for Trebše [file mmc1.pdf]

BLINDED **CONFLICT OF INTEREST STATEMENT**

American Association of Hip and Knee Surgeons

(Adopted from the American Academy of Orthopaedic Surgeons disclosure statement)

The following form **must be filled out completely listing all author affiliations**. **All items require a response. If there is no relevant disclosure for a given item, enter "*None*."**

One BLINDED Conflict of Interest form (no author names used) should be submitted per manuscript with all author disclosures.

Manuscript Title: "Global hip offset is an important factor in the success of abductor mechanism insufficiency repair after total hip arthroplasty: a case series"

1. Royalties from a company or supplier (The following conflicts were disclosed) None

2. Speakers bureau/paid presentations for a company or supplier (The following conflicts were disclosed) Biomerieux

3A. Paid employee for a company or supplier (The following conflicts were disclosed) None

3B. Paid consultant for a company or supplier (The following conflicts were disclosed) Zimmer BIomet

3C. Unpaid consultants for a company or supplier (The following conflicts were disclosed) Ceramtech

4. Stock or stock options in a company or supplier (The following conflicts were disclosed) None

5. Research support from a company or supplier as a Principal Investigator (The following conflicts were disclosed) Zimmer Biomet

6. Other financial or material support from a company or supplier (The following conflicts were disclosed) DePuy Sinthes

7. Royalties, financial or material support from publishers (The following conflicts were disclosed) Springer Verlag

8. Medical/Orthopaedic publications editorial/governing board (The following conflicts were disclosed) Journal of Bone and Joint Infections associate editor

9. Board member/committee appointments for a society (The following conflicts were disclosed) EBJIS past president

Each author must sign AND print or type his/her name, date and submit a separate form

In addition, one BLINDED Conflict of Interest form (no author names used) should be submitted per manuscript with all author disclosures.

RIHARD TREBŠE Digitalno podpisal RIHARD TREBŠE Datum: 2025.09.22 09:57:01 +02'00'

Rihard Trebše

Author Name (Print or Type)	Author Signature	Date
