# Supplementary material for: Global Hip Offset is an Important Factor in the Success of Abductor Mechanism Insufficiency Repair After Total Hip Arthroplasty: A Case Series
Source: Arthroplast Today. 2025 Oct 10;36:101861. doi: 10.1016/j.artd.2025.101861 (PMC12549797; doi:10.1016/j.artd.2025.101861)
Supplement: Conflict of Interest Statement for Mihalič [file mmc3.pdf]

BLINDED CONFLICT OF INTEREST STATEMENT

American Association of Hip and Knee Surgeons

(Adopted from the American Academy of Orthopaedic Surgeons disclosure statement)

The following form **must be filled out completely listing all author affiliations**. **All items require a response. If there is no relevant disclosure for a given item, enter "*None*."**

One BLINDED Conflict of Interest form (no author names used) should be submitted per manuscript with all author disclosures.

Manuscript Title: "Global hip offset is an important factor in the success of abductor mechanism insufficiency repair after total hip arthroplasty: a case series"

1. Royalties from a company or supplier (The following conflicts were disclosed) None

2. Speakers bureau/paid presentations for a company or supplier (The following conflicts were disclosed) None

3A. Paid employee for a company or supplier (The following conflicts were disclosed) None

3B. Paid consultant for a company or supplier (The following conflicts were disclosed) None

3C. Unpaid consultants for a company or supplier (The following conflicts were disclosed) None

4. Stock or stock options in a company or supplier (The following conflicts were disclosed) None

5. Research support from a company or supplier as a Principal Investigator (The following conflicts were disclosed)
None

6. Other financial or material support from a company or supplier (The following conflicts were disclosed) None

7. Royalties, financial or material support from publishers (The following conflicts were disclosed) None

8. Medical/Orthopaedic publications editorial/governing board (The following conflicts were disclosed) None

9. Board member/committee appointments for a society (The following conflicts were disclosed)
EFORT Board Member

Each author must sign AND print or type his/her name, date and submit a separate form

In addition, one BLINDED Conflict of Interest form (no author names used) should be submitted per manuscript with all author disclosures.

Rene Mihalič		21.09.2025
Author Name (Print or Type)	Author Signature	Date
